# Supplementary material for: Limiting oxidative DNA damage reduces microbe-induced colitis-associated colorectal cancer
Source: Nat Commun. 2020 Apr 14;11:1802. doi: 10.1038/s41467-020-15549-6 (PMC7156452; doi:10.1038/s41467-020-15549-6)
Supplement: Supplementary file 1 — Supplementary Information [file 41467_2020_15549_MOESM1_ESM.pdf]

Limiting Oxidative DNA Damage Reduces Microbe-Induced Colitis Associated Colorectal Cancer

Irrazabal et al

Supplementary Section

Supplementary Tables

Supplementary Table 1. Primers used to monitor infection by PCR.

Helicobacter genus primers	GCTATGACGGGTATCC
	GATTTTACCCCTACACCA
Helicobacter typhlonius primers	AGGGACTCTTAAATATGCTCCTAGAGT
	ATTCATCGTGTTTGAATGCGTCAA
Helicobacter mastomyrinus primers	AGAACTGCATTTGAAACTATGAG
	CAGTATTGCGTCTCTTTGTA
Helicobacter hepaticus primers	GAAACTGTTACTCTG
	TCAAGCTCCCCGAAGGG
Escherichia coli NC101 (*clbB*) primers [18]	GCAACATACTCGCCCAGCT
	TCTCAAGGCGTTGTTGTTTG
Enterotoxigenic *Bacteroides fragilis* primers	GTACACACCGCCCGT
	AATTTAGAACCAATGAACG

Supplementary Table 2. Primers used to monitor infection by qPCR.

Helicobacter genus–specific primers	ACCAAGGC(A/T)ATGACGGGTATC
	CGGAGTTAGCCGGTGCTTATT
Citrobacter rodentium primers	ATGCCGCAGATGAGACAGTTG
	CGTCAGCAGCCTTTTCAGCTA
Eubacterium genus primers	ACTCCTACGGGAGGCAGCAGT
	ATTACCGCGGCTGCTGGC

Supplementary Table 3. Primers used to analyze inflammatory signaling by qPCR.

IFN-γ	GATATCTGGAGGAACTGGCAAAA
	CTTCAAAGAGTCTGAGGTAGAAAGAGATAAT
TNF-α	AATGGCCTCCCTCTCATCAGTT
	CCACTTGGTGGTTTGCTACGA
IL-1β	CTCCACCTCAATGGACAGAA
	GCCGTCTTTCATTACACAGG

IL-12	CACCCTTGCCCTCCTAAACC
	CAAGGCACAGGGTCATCATC
IL-6	GAGGATACCACTCCCAACAGACC
	AAGTGCATCATCGTTGTTCATACA
IL-17	TCTCCACCGCAATGAAGACC
	CACACCCACCAGCATCTTCT

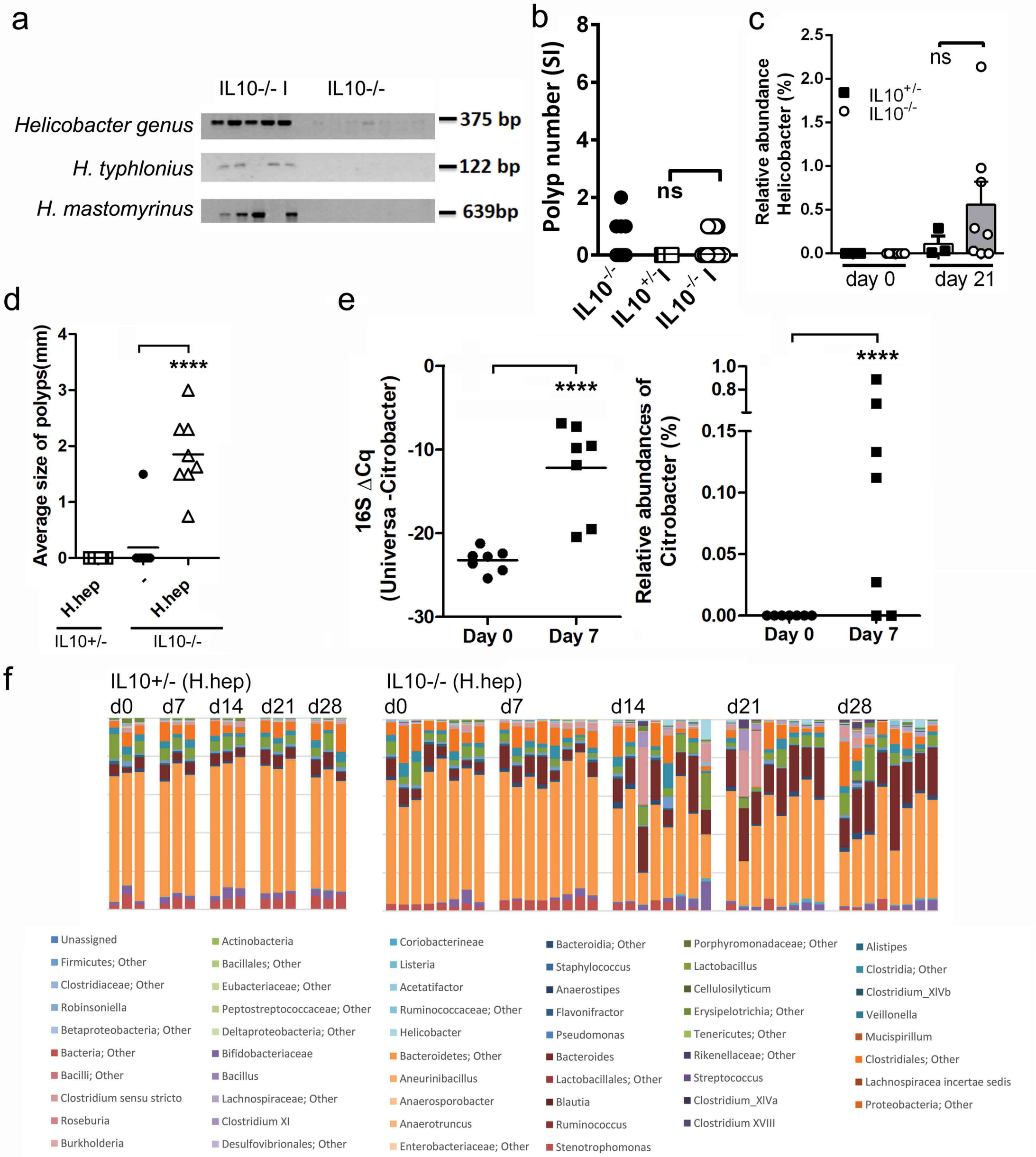

Supplementary Fig. 1. Not all bacterial colon infections trigger irreversible dysbiosis, and colon tumorigenesis in IL10-/- mice. (a) Infection of IL10-/- mice with H. typhlonius and H. mastomyrinus (IL10-/-I) was confirmed by PCR. Image representative of three independent experiments. (b) Polyp number was analyzed in the small intestine of 9-week old Helicobacter infected (IL10-/- and IL10+/- littermates) or uninfected IL10-/- mice. N=35 mice were examined. (c) H. hepaticus infection in IL10+/- and IL10-/- mice was confirmed using 16S rRNA gene sequencing analysis. N=11 mice were examined. Data are presented as mean values + SEM. (d) Average polyp size in mice of the indicated genotypes, infected with H. hepaticus. Each symbol represents the average tumor size for one mouse. N=24 mice were examined. (e) Relative abundance of C. rodentium was evaluated by 16S rRNA gene qPCR at day 0 and 7 post challenge infection. N=14 mice were examined. (f) Bar chart depicting the relative proportions of the bacterial genus present in the indicated mice before and after infection with H. hepaticus (d= day). When genus was not identified, the lowest taxon identified was depicted. Each bar represents data of a single mouse. Data was derived from the 16S rRNA gene sequencing. Data in b-e were analyzed using the two-sided non-parametric T-test Mann-Whitney. ****p<0.0001, ns= nonsignificant.

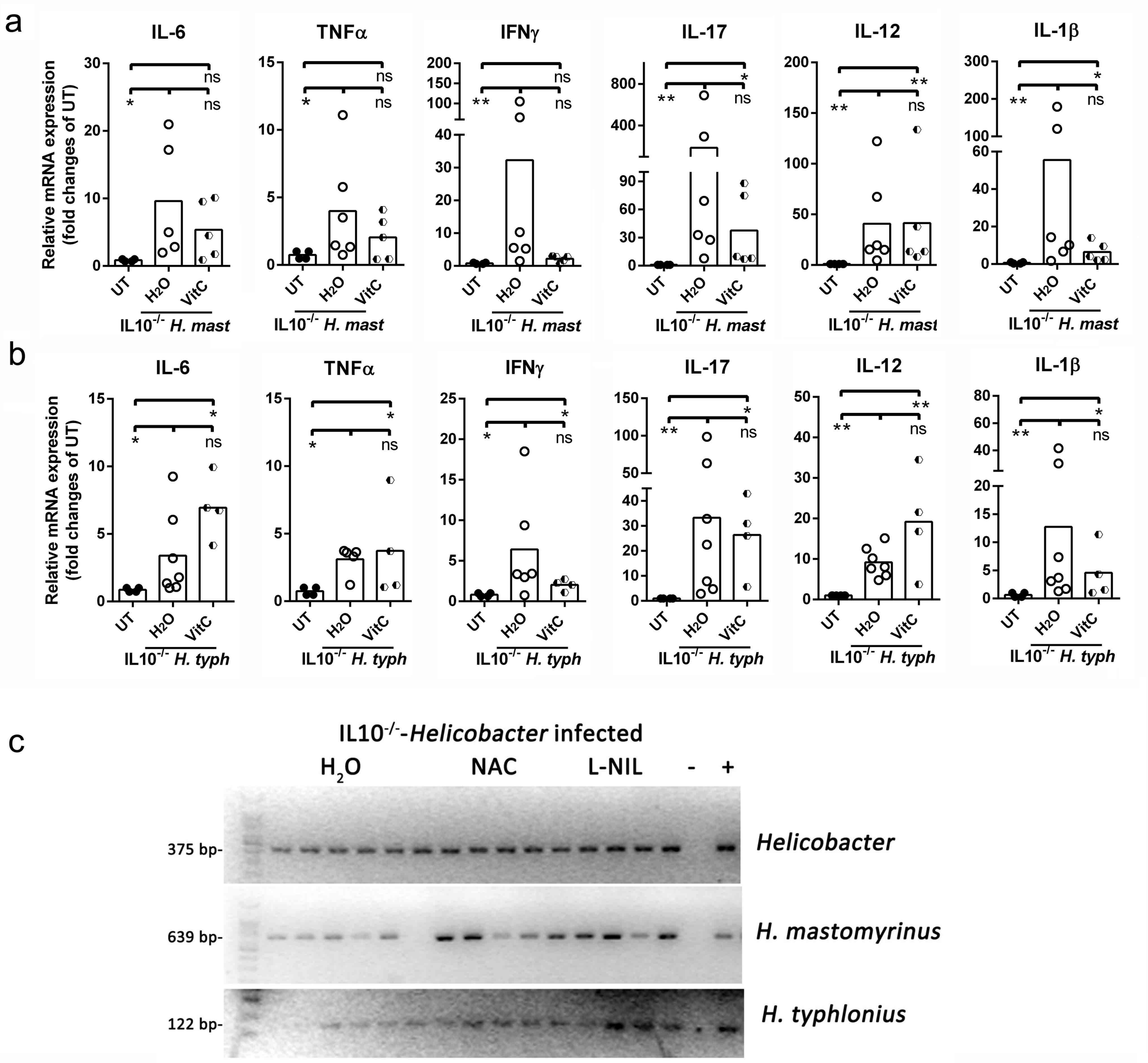

Supplementary Fig. 2. L-NIL and antioxidants do not affect Helicobacter colonization or inflammatory cytokine expression in the colon of IL10-/- mice. (a) cDNA levels of the indicated inflammatory cytokines were quantified by qPCR in H. mastomyrinus-infected mice that have received the indicated treatments. Relative mRNA expression was normalized to 1 for untreated IL10-/- mice. N=15 mice were examined. (b) Same as a, except H. typhlonius-infected IL10-/- mice were used. N=15 mice were examined. (c) Infection with H. typhlonius and H. mastomyrinus of IL10-/- mice treated with antioxidants and L-NIL was confirmed by PCR. Image representative of three independent experiments. Data in a and b were analyzed using the two-sided non-parametric T-test Mann-Whitney. *$p<0.05$, **$p<0.01$, ns= nonsignificant.

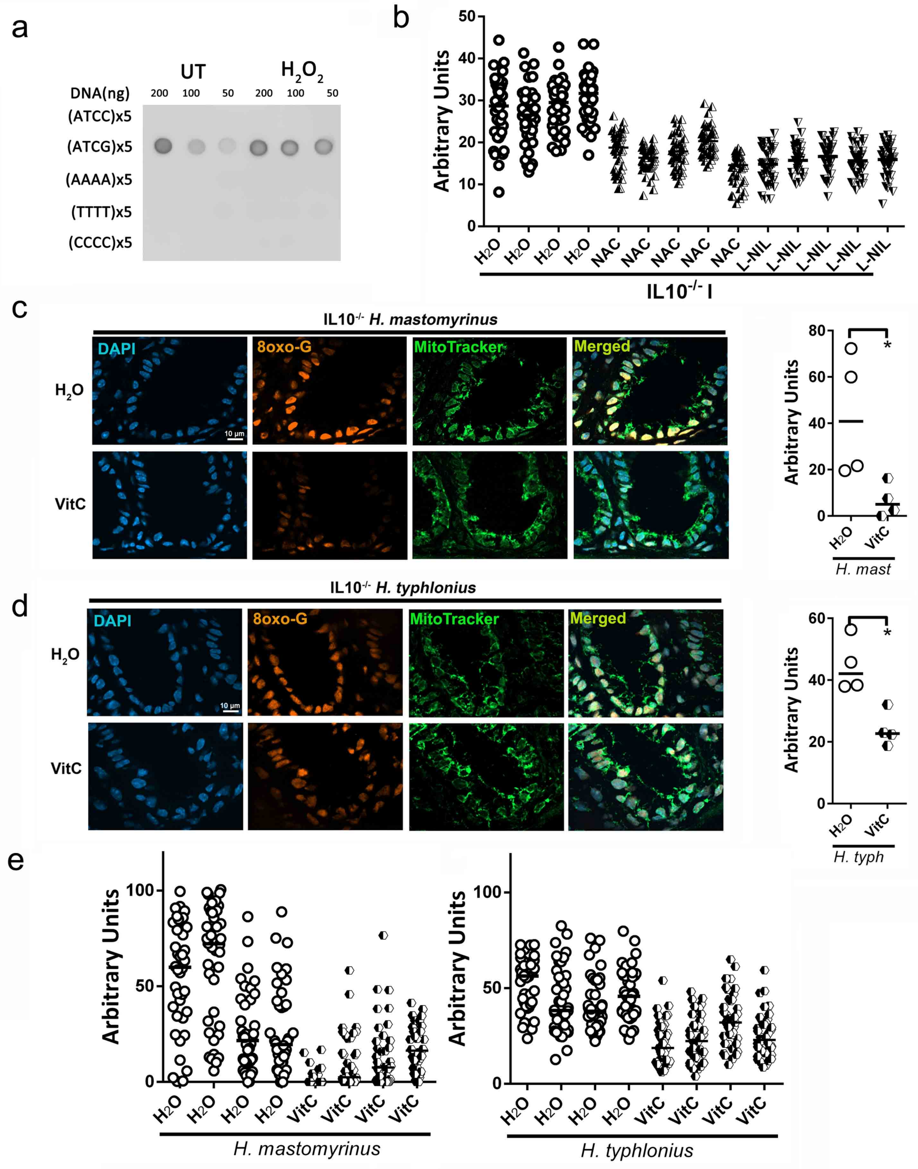

Supplementary Fig. 3. L-NIL and antioxidants reduce 8-oxoG in the inflamed colon of Helicobacter-infected IL10-/- mice. (a) Testing the specificity of the anti-8-oxoG antibody. Several oligonucleotides with the indicated sequence were oxidized with H2O2 or left as is, and a dot blot assay was used to determine 8-oxoG antibody binding strength. The data shows that the anti-8-oxoG antibody only binds to DNA containing G nucleotides, and this binding is enhanced by H2O2 treatment. Image representative of four independent experiments. (b) ImageJ quantification of 8-oxoG immunofluorescence of Helicobacter-infected IL10-/- I mice shown in Fig. 2e and others not shown. One dot represents the intensity of fluorescence of one nucleus per mouse. 10 nuclei per field, and 4 field per mouse were analyzed. N=14 mice were examined. (c) Immunofluorescence for 8-oxoG and MitoTracker in colon from H. Mastomyrinus-infected IL10-/- mice untreated or treated with VitC. Magnification 100X. Scale bar=10 µm. Right panel: One Dot represents the median intensity of fluorescence of 40 nuclei per mouse. N=8 biologically independent samples were examined over four independent experiments. (d) Same as c, except H. typhlonius-infected IL10-/- mice administered with VitC were used. N=8 biologically independent samples were examined over four independent experiments. (e) Left panel: ImageJ quantification of 8-oxoG immunofluorescence of H. mastomyrinus infected IL10-/- mice shown in Supplementary Fig. 3c. One dot represents the intensity of fluorescence of one nucleus per mouse. 10 nuclei per field, and 4 field per mouse were analyzed. Right panel: Same as left panel, except that H. typhlonius infected IL10-/- mice shown in Supplementary Fig. 3d were used. N=16 mice were examined. Data in c and d were analyzed using the two-sided non-parametric T-test Mann-Whitney.*p<0.05.

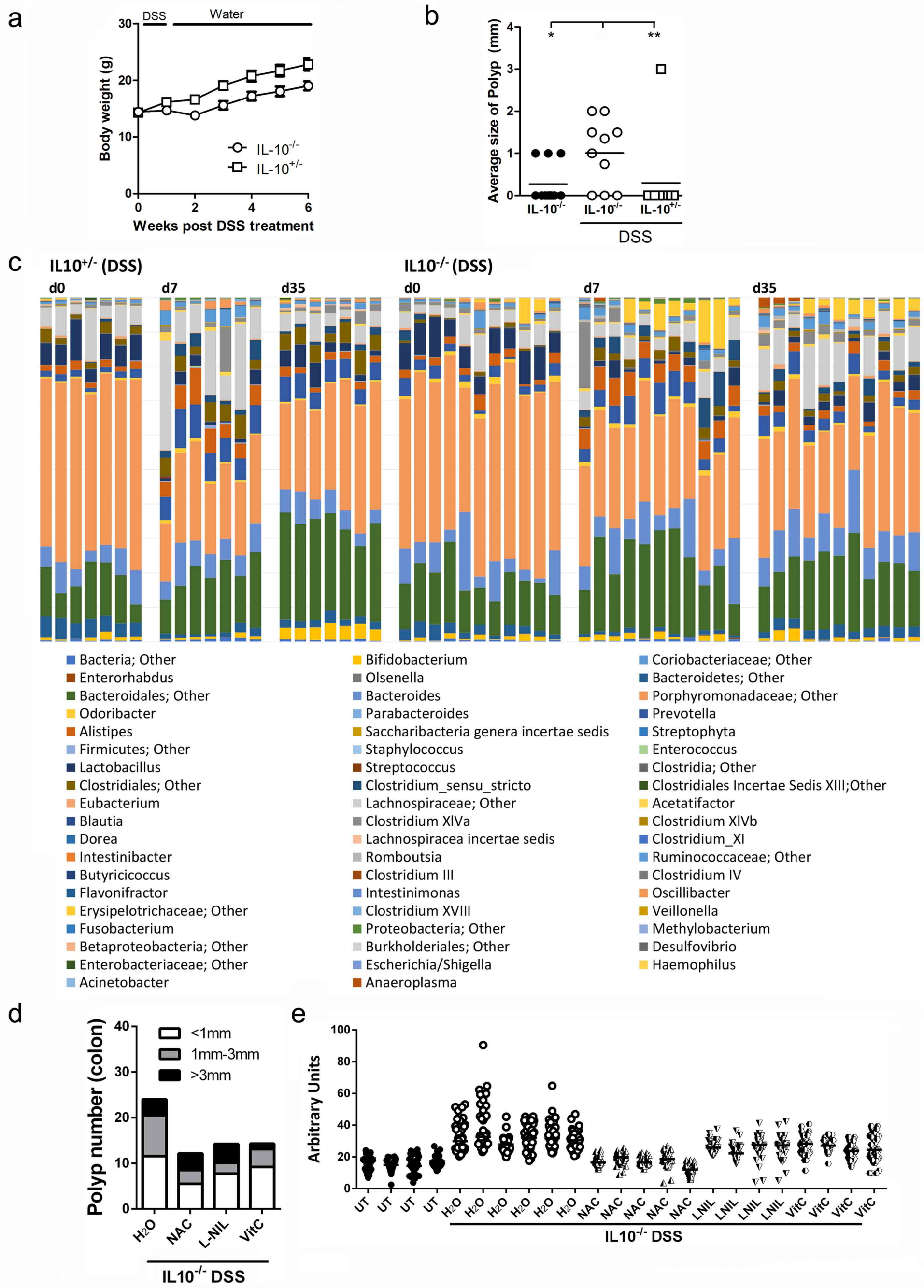

Supplementary Fig. 4. A single DSS challenge can trigger colitis, dysbiosis and colon tumorigenesis in a genetically susceptible host, and antioxidants revert tumorigenesis. (a) 4-week-old IL10+/- and IL10-/- mice were treated with 1%DSS and body weight was monitored for 6-weeks. N=20 mice were examined. Data are presented as mean values +/- SEM. . (b) Average polyp size in 10-week old mice of the indicated genotypes, treated or untreated with 1% DSS. Each symbol represents the average tumor size for one mouse. N=26 mice were examined. (c) Bar chart depicting the relative proportions of the bacterial genus present in the indicated mice before and after treatment with 1% DSS (d= day). When genus was not identified, the lowest taxon identified was depicted and genus was defined as “Other”. Each bar represents data from a single mouse. Data was derived from the 16S rRNA gene sequencing. (d) Polyps counted on Figure 4d were graded according their size. (e) 4-week-old IL10-/- mice were treated with 1% DSS for one week and then treated with L-NIL, NAC, or VitC for 4 weeks. ImageJ quantification of 8-oxoG immunofluorescence of images shown in Fig. 4e and others not shown. One dot represents the intensity of fluorescence of one nucleus per mouse. 10 nuclei per field, and 4 field per mouse were analyzed. N=23 mice were examined. Data in b was analyzed using the two-sided non-parametric T-test Mann-Whitney. *p<0.05, **p<0.01. Error bars represent SEM.

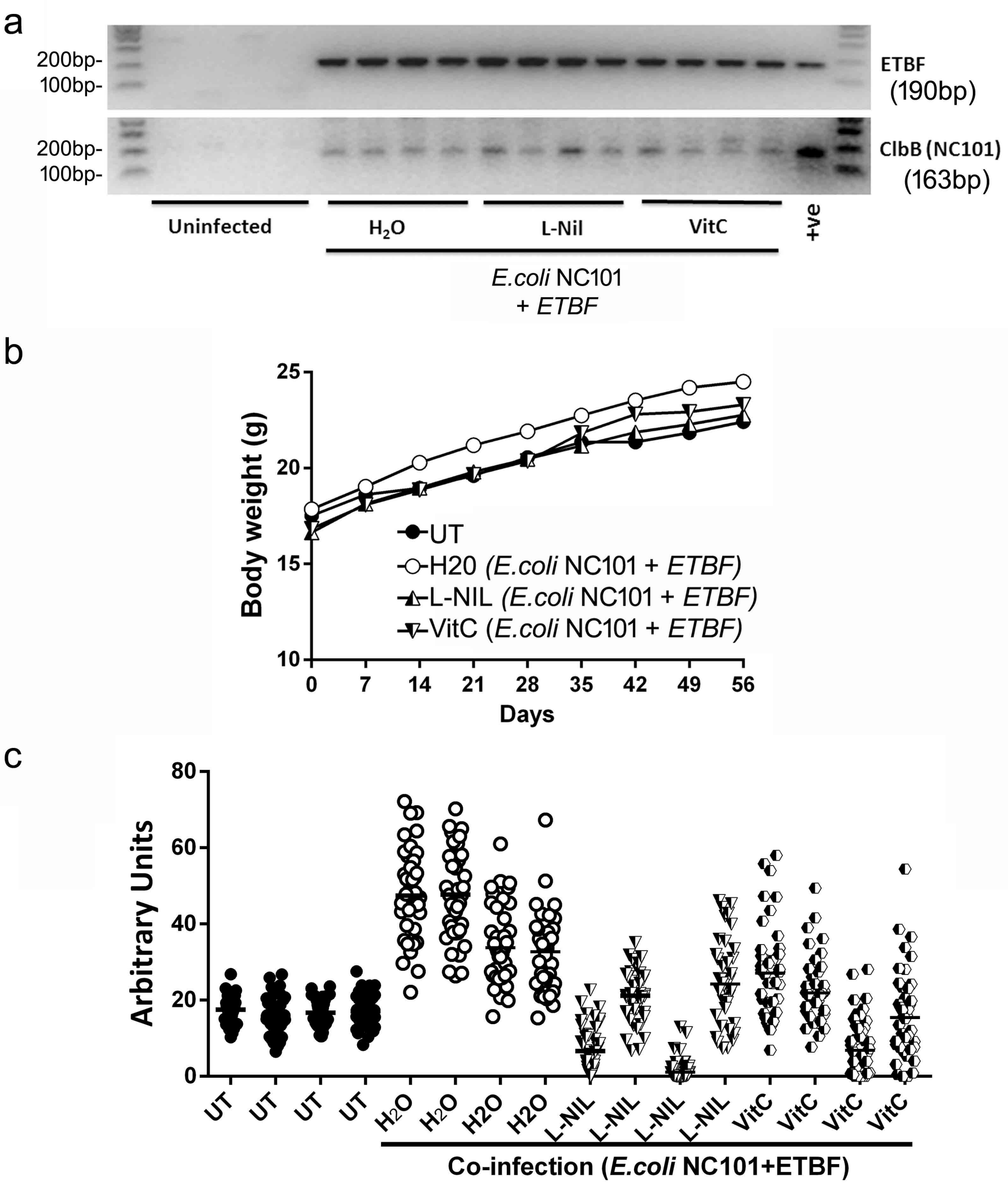

Supplementary Fig. 5. L-NIL and VitC do not affect inflammation or co-colonization of E.coli NC101 and ETBF in IL10-/- mice, but reduce oxidative DNA damage in colon epithelial cells. (a) Co-colonization with E. coli NC101 and ETBF in IL10-/- mice was confirmed by PCR. Image representative of three independent experiments. (b) The weight of IL10-/- mice unifected (UT), infected with E. coli NC101 and ETBF-infected, or treated with L-NIL or VitC was measured. N=24 mice were examined. Dots represent the median weight per group. (c) ImageJ quantification of 8-oxoG immunofluorescence of images shown in Fig. 5f and others not shown. One dot represents the intensity of fluorescence of one nucleus per mouse. 10 nuclei per field, and 4 field per mouse were analyzed. N=16 mice were examined.

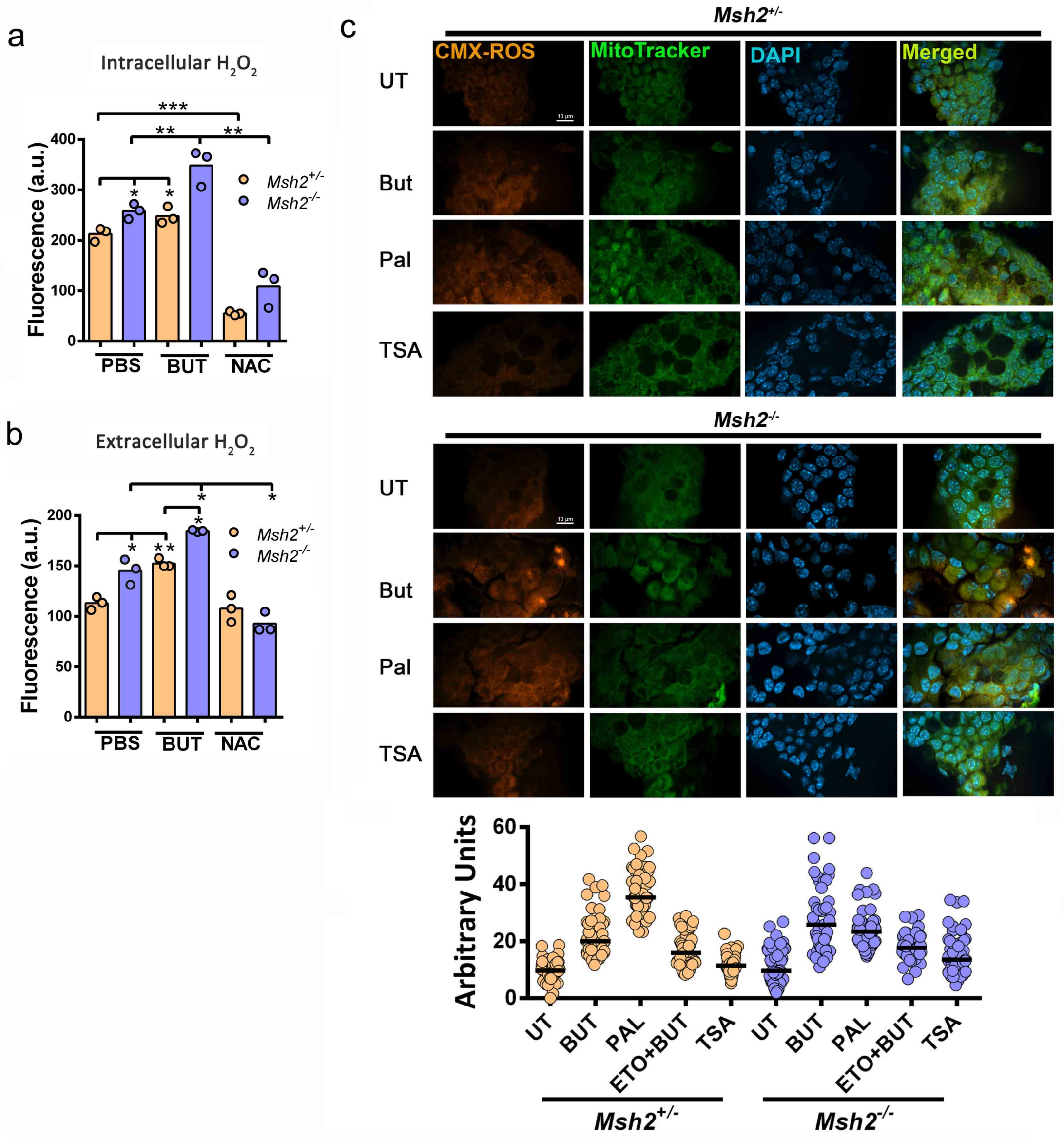

Supplementary Fig. 6. Butyrate induces ROS colon in epithelial cells. (a) CM-DCFDA was used to measure intracellular H2O2 in freshly extracted colonocytes from the indicated genotypes. Colonocytes were treated with 2 mM Butyrate or 0.1% NAC. N=3 biologically independent samples were examined over three independent experiments. (b) Amplex Red was used to measure extracellular H2O2 in freshly extracted colonocytes from the indicated genotypes. Colonocytes were treated with 0.5 mM butyrate or 0.1% NAC. N=3 biologically independent samples were examined over three independent experiments. (c) Butyrate metabolism induces ROS in colon epithelial crypts. Butyrate-depleted colon crypts were treated with 0.5 mM butyrate (But), 100 μM palmitate (Pal), 2μM TSA or left untreated (UT). Metabolically active mitochondria were stained with Mitotracker Red CMXRos, while all mitochondria were stained with Mitotracker FM and analyzed by immunofluorescence. Scale bar=10 μm. Lower panel shows the intensity of fluorescence of CMXRos in 15 cells per field, and three fields per treatment analyzed using ImageJ. N=4 biologically independent samples were examined over two independent experiments. Data in a and b were analyzed using the two-sided T-test. *$p<0.05$, **$p<0.01$, ***$p<0.001$.

a

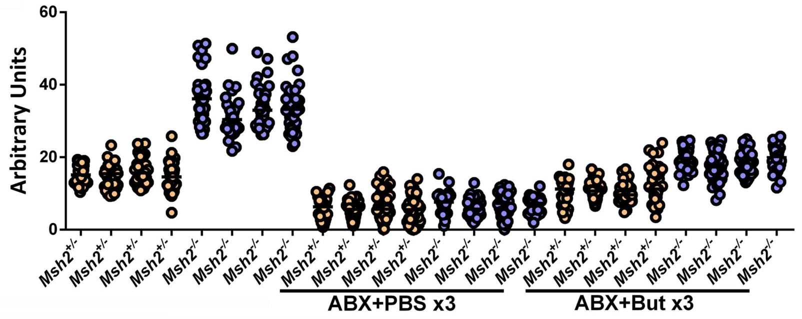

b

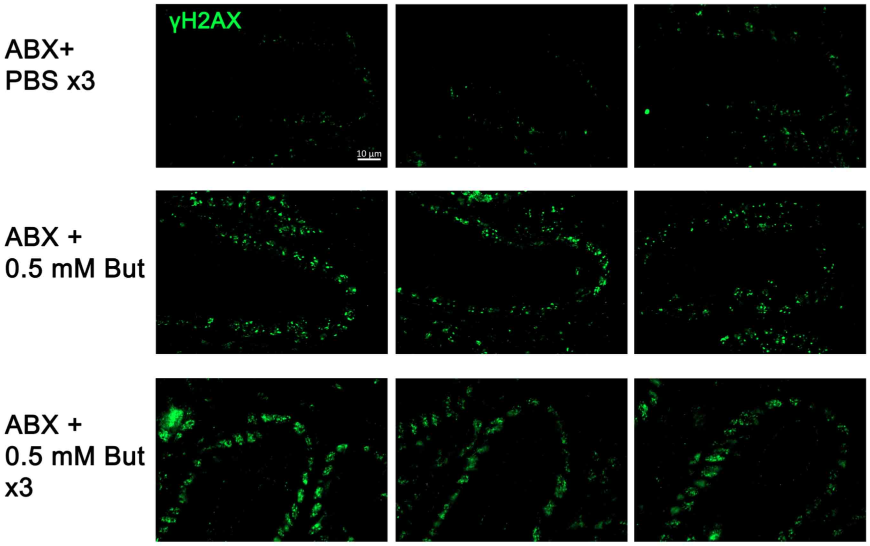

Supplementary Fig. 7. Microbiota and butyrate induce oxidative DNA damage and double-stranded DNA breaks in Msh2-/- colon epithelial cells. (a) ImageJ quantification of 8-oxoG immunofluorescence of images shown in Fig. 6a. One dot represents the intensity of fluorescence of one nucleus per mouse. 10 nuclei per field, and 4 field per mouse were analyzed. N=24 mice were examined. (b) Representative images for γH2AX immunofluorescence in colon from Msh2-/- mice at the indicated treatments (n=3). Magnification 100X. Scale bar=10 μm.

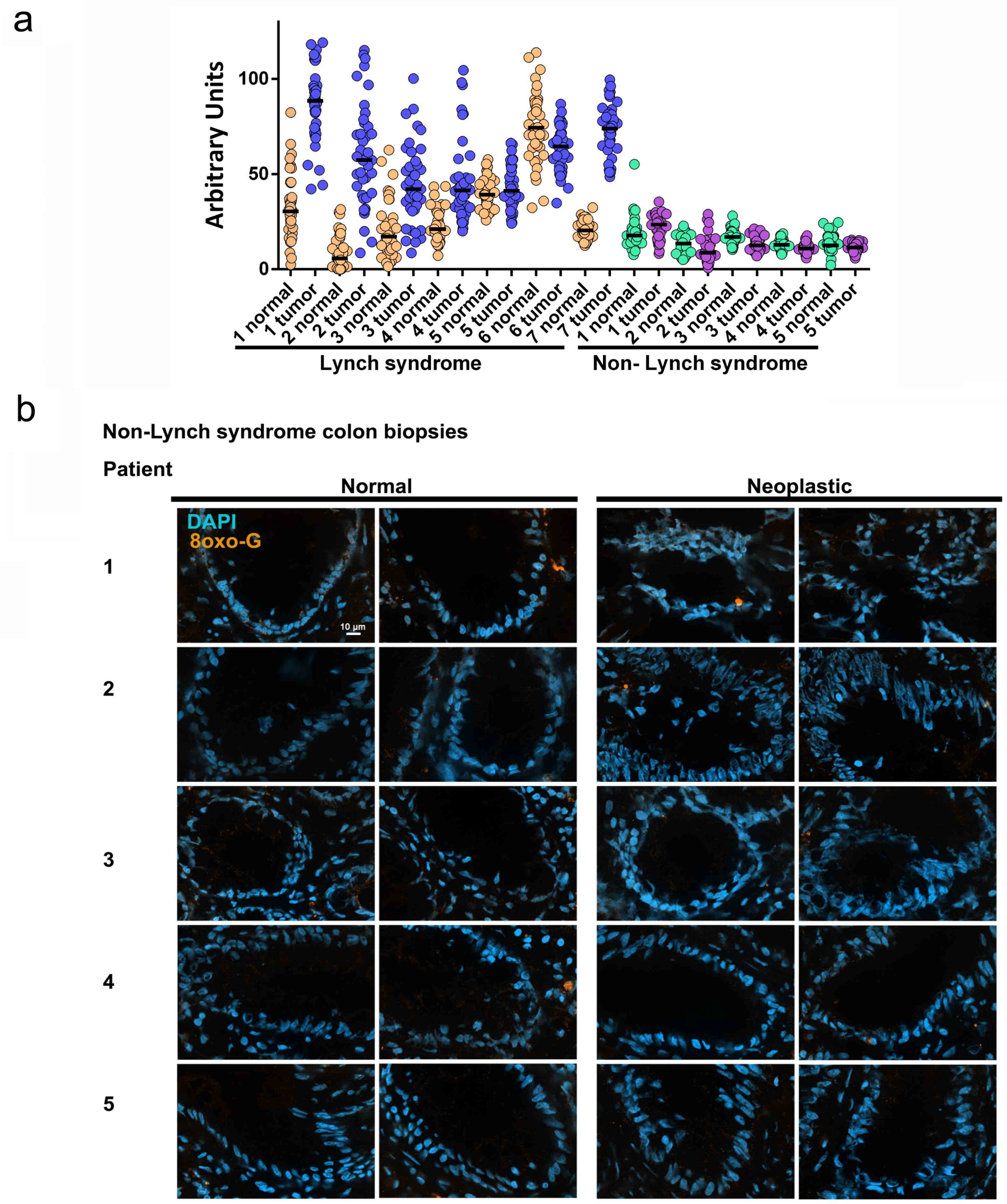

Supplementary Fig. 8. Non-Lynch patients have normal levels of 8-oxoG in the colon. (a) ImageJ quantification of 8-oxoG immunofluorescence of images shown in Fig. 6b and others not shown. One dot represents the intensity of fluorescence of one nucleus per sample . 10 nuclei per field, and 4 fields per patient were analyzed. N=12 biologically independent samples (plus 12 matched normal tissue) were examined over two independent experiments. (b) Immunofluorescence for DAPI and 8-oxoG in neoplastic and normal flanking colon from five non-Lynch syndrome patients. Representative images of two independent experiments are depicted. Scale bar=10 µm. Data is summarized in Fig. 6b, right panel.

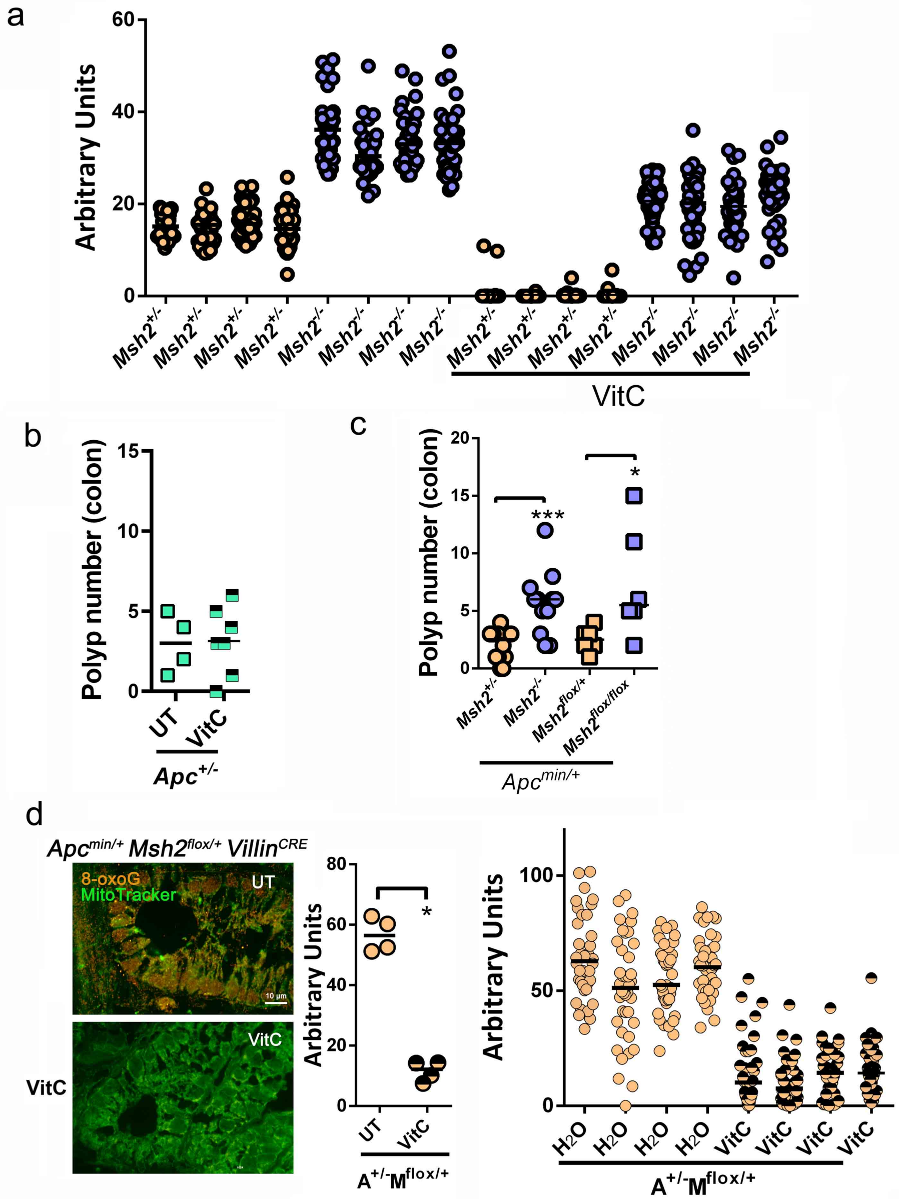

Supplementary Fig. 9. Antioxidants reduce 8-oxoG in Msh2-deficient mice but not tumorigenesis. (a) ImageJ quantification of 8-oxoG immunofluorescence of images shown in Fig. 7e and other not shown. One dot represents the intensity of fluorescence of one nucleus per mouse. 10 nuclei per field, and four field per mouse were analyzed. N=16 mice were examined. (b) 4-week old Apcmin/+ mice were untreated or treated with VitC for 12-weeks, and polyp number were reported in the colon of 16-week old mice. N=11 mice were examined. (c) Polyp number were analyzed in the colon of 6-week old mice of the indicated genotypes. The recombinase CRE is under the control of Villin, and therefore in Msh2flox/flox mice Msh2 is only deleted in intestinal epithelial cells. Each symbol represents the numbers of polyps in one mouse. N=32 mice were examined. (d) Left panel: Immunofluorescence for 8-oxoG and MitoTracker in nondysplastic colon from Apcmin/+ Msh2flox/+ villin CRE mice at the indicated treatments. Magnification 100X. Scale bar=10 µm. Middle panel: quantification of 8-oxoG immunofluorescence. One dot represents the median intensity of fluorescence of 40 nuclei per mouse as shown in right panel. Right panel: One dot represents the intensity of fluorescence of one nucleus per mouse. 10 nuclei per field, and four field per mouse were analyzed. N=8 mice were examined. Data in c and d were analyzed using the two-sided non-parametric T-test Mann-Whitney.*$p<0.05$, ***$p<0.001$.
