# Supplementary material for: Limiting oxidative DNA damage reduces microbe-induced colitis-associated colorectal cancer
Source: Nat Commun. 2020 Apr 14;11:1802. doi: 10.1038/s41467-020-15549-6 (PMC7156452; doi:10.1038/s41467-020-15549-6)
Supplement: Supplementary file 3 — Reporting Summary [file 41467_2020_15549_MOESM3_ESM.pdf]

nature research

Corresponding author(s): Alberto Martin

Last updated by author(s): Mar 3, 2020

Reporting Summary

Nature Research wishes to improve the reproducibility of the work that we publish. This form provides structure for consistency and transparency in reporting. For further information on Nature Research policies, see Authors & Referees and the Editorial Policy Checklist .

Statistics

For all statistical analyses, confirm that the following items are present in the figure legend, table legend, main text, or Methods section.

n/a	Confirmed	
☐	☒	The exact sample size (*n*) for each experimental group/condition, given as a discrete number and unit of measurement
☐	☒	A statement on whether measurements were taken from distinct samples or whether the same sample was measured repeatedly
☐	☒	The statistical test(s) used AND whether they are one- or two-sided<br>*Only common tests should be described solely by name; describe more complex techniques in the Methods section.*
☐	☒	A description of all covariates tested
☒	☐	A description of any assumptions or corrections, such as tests of normality and adjustment for multiple comparisons
☐	☒	A full description of the statistical parameters including central tendency (e.g. means) or other basic estimates (e.g. regression coefficient) AND variation (e.g. standard deviation) or associated estimates of uncertainty (e.g. confidence intervals)
☐	☒	For null hypothesis testing, the test statistic (e.g. *F*, *t*, *r*) with confidence intervals, effect sizes, degrees of freedom and *P* value noted<br>*Give P values as exact values whenever suitable.*
☒	☐	For Bayesian analysis, information on the choice of priors and Markov chain Monte Carlo settings
☒	☐	For hierarchical and complex designs, identification of the appropriate level for tests and full reporting of outcomes
☒	☐	Estimates of effect sizes (e.g. Cohen's *d*, Pearson's *r*), indicating how they were calculated

Our web collection on statistics for biologists contains articles on many of the points above.

Software and code

Policy information about availability of computer code

Data collection	No software was used
Data analysis	Graphpad Prism 6 was used for all statistical data analysis. USEARCH version 9.2 and vsearch were used for analysis and process 16S rRNA sequences. Intensity of fluorescence was analyzed using ImageJ.

For manuscripts utilizing custom algorithms or software that are central to the research but not yet described in published literature, software must be made available to editors/reviewers. We strongly encourage code deposition in a community repository (e.g. GitHub). See the Nature Research guidelines for submitting code & software for further information.

Data

Policy information about availability of data

All manuscripts must include a data availability statement. This statement should provide the following information, where applicable:

- Accession codes, unique identifiers, or web links for publicly available datasets
- A list of figures that have associated raw data
- A description of any restrictions on data availability

The data that support the findings of this study are available from the corresponding author upon reasonable request.
Figures with associated raw data:
Figure 1-7.
Figure S1b-e; S2 a,b; S3b-e; S4; S5b-c; S6; S7a; S8a; S9.

October 2018

Field-specific reporting

Please select the one below that is the best fit for your research. If you are not sure, read the appropriate sections before making your selection.

- [x] Life sciences
- [ ] Behavioural & social sciences
- [ ] Ecological, evolutionary & environmental sciences

For a reference copy of the document with all sections, see nature.com/documents/nr-reporting-summary-flat.pdf

Life sciences study design

All studies must disclose on these points even when the disclosure is negative.

Sample size	No sample size calculations were made. Sample sizes for experiments were based on the numbers of mice available for each experiment, and were generally above 8 mice per group.
Data exclusions	No data was excluded from this manuscript
Replication	All experiments were independently replicated two to four times and were reproducible. Some experiments were replicated using different assays validating the conclusions.
Randomization	Groups of mice were randomly treated with antioxidants or left untreated such that each group would have similar numbers of mice. Samples associated to 12 colorectal cancer patients (7 Lynch syndrome and 5 non-Lynch syndrome) were randomly provided by clinicians.
Blinding	Each mice was assigned an ID unrelated to their treatment. All samples generated from a determined mouse have this ID, and therefore slides for histology, immunofluorescence and fecal samples were analyzed in a blinded manner. Clinical samples, also have and ID and were analyzed in a blinded manner. The pathologist was blinded when analyzing the slides of mouse colons, as indicated in the manuscript.

Reporting for specific materials, systems and methods

We require information from authors about some types of materials, experimental systems and methods used in many studies. Here, indicate whether each material, system or method listed is relevant to your study. If you are not sure if a list item applies to your research, read the appropriate section before selecting a response.

Materials & experimental systems

n/a	Involved in the study	
[ ]	[x]	Antibodies
[x]	[ ]	Eukaryotic cell lines
[x]	[ ]	Palaeontology
[ ]	[x]	Animals and other organisms
[ ]	[x]	Human research participants
[x]	[ ]	Clinical data

Methods

n/a	Involved in the study	
[x]	[ ]	ChIP-seq
[x]	[ ]	Flow cytometry
[x]	[ ]	MRI-based neuroimaging

Antibodies

Antibodies used	8-oxoG antibody: DNA and RNA Oxidative Damage Markers Monoclonal Antibody; QED Bioscience; catalog number: 12501; lot: 102411; clone 15A3; 1:100 dilution for immunofluorescence and 1:1000 for dot blot.<br>PE-tagged anti mouse IgG2b antibody; BioLegend; catalog number: 406708; clone RMG2b-1; 1:400 dilution.<br>anti-γH2AX Ser139 antibody; EMD Millipore; catalog number: 05-636; lot: 2854120; clone JBW301; 1:100 dilution.<br>Alexa488-tagged anti mouse IgG1 antibody; Thermo Fisher Scientific, catalog number: A21121; lot: 1820808; 1:400 dilution.<br>HRP-tagged goat anti mouse antibody; Southern Biotech; catalog number:1036-05; 1:4000 dilution.
Validation	To validate anti 8-oxoG antibody specificity, five oligonucleotides: ATCGx5, ATCCx5, AAAAx5, TTTTx5 and CCCCx5 were treated with H2O2 and blotted in serial dilutions on a Nitrocellulose membrane. The membrane was exposed to UV light for 5min to crosslink the oligonucleotides, and blocked by incubation with 10%BSA for 1h at RT. After washing with PBS, the membrane was incubated with anti 8-oxoG antibody (1:1000) for 1h at RT, followed by incubation with an HRP-tagged goat anti mouse antibody (Southern Biotech, 1:4000). The membrane was incubated with Clarity Western ECL (Biorad) and luminescence was detected with a Microchemi 4.2 Bioimaging system (DNR).<br>This antibody recognizes markers of oxidative damage in DNA and RNA. We have also confirmed that pretreatment of colon tissue samples with RNase reduce cytoplasmic staining.<br>References<br>Park et al. (1992) Assay of excised oxidative DNA lesions: Isolation of 8-oxoguanine and its nucleoside derivatives from biological fluids with a monoclonal antibody column. Proc Natl Acad Sci (USA) 89: 3375- 3379.<br>Nunomura et al. (1999) RNA Oxidation is a prominent feature of vulnerable neurons in Alzheimer's Disease. J Neuroscience 19:

1959-1964.
Cui et al. (1999) Oxidative damage to the c-fos gene and reduction of its transcription after focal cerebral ischemia. J Neurochemistry 73:1164-1174.
Salganik et al. (2000) Dietary antioxidant depletion: enhancement of tumor apoptosis and inhibition of brain tumor growth in transgenic mice. Carcinogenesis 21: 909-914.
Tanaka et al. (2007) Oxidized messenger RNA induces translation errors. Proc Natl Acad Sci (USA) 104: 66-71.
Zhan et al. (2015) Localized control of oxidized RNA. Journal of Cell Science 128: 4210-4219.
Kharel et al. (2016) Evidence of extensive RNA oxidation in normal appearing cortex of multiple sclerosis brain. Neurochemistry International 92: 43-48.

We have previously validated anti γ-H2AX antibody by irradiating cells. Non-irradiated cells do no have a significant number of γH2AX foci while irradiation induces γH2AX foci in a dose dependent manner.
Manufacturer validation statement: Anti-phospho-Histone H2A.X (Ser139), clone JBW301 is a well published Mouse Monoclonal Antibody validated in ChIP, ICC, IF, WB. This purified mAb is highly specific for phospho-Histone H2A.X (Ser139) also known as H2AXS139p.
References:
Limiting replication stress during somatic cell reprogramming reduces genomic instability in induced pluripotent stem cells.
Ruiz, S; Lopez-Contreras, AJ; Gabut, M; Marion, RM; Gutierrez-Martinez, P; Bua, S; Ramirez, O; Olalde, I; Rodrigo-Perez, S; Li, H; Marques-Bonet, T; Serrano, M; Blasco, MA; Batada, NN; Fernandez-Capetillo, O
Nature communications 6 8036 2015
Highly multiplexed imaging of single cells using a high-throughput cyclic immunofluorescence method.
Lin, JR; Fallahi-Sichani, M; Sorger, PK
Nature communications 6 8390 2015

Animals and other organisms

Policy information about studies involving animals; ARRIVE guidelines recommended for reporting animal research

Laboratory animals	All strains were in the Blck/6 background and were reported in the Methods section. Male and female mice were used. Experiments started when mice were 3-4 weeks of age, and finished at different times depending on the experiment but no later than 16 weeks.
Wild animals	No wild animals were used in this study.
Field-collected samples	No field-collected samples were used in this study.
Ethics oversight	All experiments using animals were approved by the University of Toronto - University Animal Care Committee (protocol 20011472)

Note that full information on the approval of the study protocol must also be provided in the manuscript.

Human research participants

Policy information about studies involving human research participants

Population characteristics	Frozen human colorectal cancer samples belong to the following population: Colorectal cancer patients; 7 Lynch syndrome patients, with mutations in MSH2 or PMS2; 5 Non- Lynch syndrome patients, which do not have mutations in MMR genes; age: 29.2 – 82; male and female.
Recruitment	No patients were recruited to this study. Frozen human colorectal cancer samples were obtained from the LTRI-Biospecimen Repository and Processing Lab.
Ethics oversight	All experiments using human tissue were approved by the University of Toronto Human Ethics panel (protocol 37296)

Note that full information on the approval of the study protocol must also be provided in the manuscript.
